# Adverse Childhood Experiences, Genetic Susceptibility, and the Risk of Osteoporosis: A Cohort Study

**DOI:** 10.3390/medicina61081387

**Published:** 2025-07-30

**Authors:** Yanling Shu, Chao Tu, Yunyun Liu, Lulu Song, Youjie Wang, Mingyang Wu

**Affiliations:** 1 Ministry of Education Key Laboratory of Environment and Health, and State Key Laboratory of Environmental Health (Incubating), School of Public Health, Tongji Medical College, Huazhong University of Science and Technology, No. 13 Hangkong Road, Wuhan 430030, China; shushu@hust.edu.cn (Y.S.); song_lulu@hust.edu.cn (L.S.); wangyoujie@mails.tjmu.edu.cn (Y.W.); 2 Department of Maternal and Child Health, School of Public Health, Tongji Medical College, Huazhong University of Science and Technology, No. 13 Hangkong Road, Wuhan 430030, China; 3 Department of Orthopaedics, The Second Xiangya Hospital of Central South University, Changsha 410011, China; tuchao@csu.edu.cn; 4 Hunan Key Laboratory of Tumor Models and Individualized Medicine, Hunan Engineering Research Center of AI Medical Equipment, The Second Xiangya Hospital of Central South University, Changsha 410011, China; 5 School of Medical Technology, Jiangsu College of Nursing, Huai’an 223001, China; liuyunyun2022@jscn.edu.cn; 6 Department of Maternal and Child Health, Xiangya School of Public Health, Central South University, No. 172 Tongzipo Road, Changsha 410013, China

**Keywords:** adverse childhood experiences, osteoporosis, genetic risk, UK Biobank, prospective cohort study

## Abstract

*Background and Objectives:* Emerging evidence indicates that individuals exposed to adverse childhood experiences (ACEs) face elevated risks for various chronic illnesses. However, the association between ACEs and osteoporosis risk remains underexplored, particularly regarding potential modifications by genetic susceptibility. This prospective cohort study aims to examine the relationship of ACEs with incident osteoporosis and investigate interactions with polygenic risk score (PRS). *Materials and Methods:* This study analyzed 124,789 UK Biobank participants initially free of osteoporosis. Cumulative ACE burden (emotional neglect, emotional abuse, physical neglect, physical abuse, sexual abuse) was ascertained through validated questionnaires. Multivariable-adjusted Cox proportional hazards models assessed osteoporosis risk during a median follow-up of 12.8 years. Moderation analysis examined genetic susceptibility interactions using a standardized PRS incorporating osteoporosis-related SNPs. *Results:* Among 2474 incident osteoporosis cases, cumulative ACEs showed dose–response associations with osteoporosis risk (adjusted hazard ratio [HR]_per one-unit increase_ = 1.07, 95% confidence interval [CI] 1.04–1.11; high ACEs [≥3 types] vs. none: HR = 1.26, 1.10–1.43). Specifically, emotional neglect (HR = 1.14, 1.04–1.25), emotional abuse (HR = 1.14, 1.03–1.27), physical abuse (HR = 1.17, 1.05–1.30), and sexual abuse (HR = 1.15, 1.01–1.31) demonstrated comparable effect sizes. Sex-stratified analysis revealed stronger associations in women. Joint exposure to high ACEs/high PRS tripled osteoporosis risk (HR = 3.04, 2.46–3.76 vs. low ACEs/low PRS) although G × E interaction was nonsignificant (P-interaction = 0.10). *Conclusions:* These results suggest that ACEs conferred incremental osteoporosis risk independent of genetic predisposition. These findings support the inclusion of ACE screening in osteoporosis prevention strategies and highlight the need for targeted bone health interventions for youth exposed to ACEs.

## 1. Introduction

Osteoporosis, characterized by compromised bone strength that predisposes individuals to fragility fractures, has emerged as a critical public health challenge with a rising prevalence among aging populations [1,2]. Globally, over 41.5 million individuals suffered from osteoporosis in 2019, and this figure is expected to climb dramatically to 263.2 million between 2030 and 2034 [3,4]. While traditional risk factors (e.g., hormonal deficiency, sedentary lifestyle) are well-established [2], emerging evidence underscores the “developmental origins of osteoporosis” hypothesis, positing that early-life insults may program lifelong skeletal fragility [5,6]. During childhood and adolescence—a critical window for achieving peak bone mass—nutritional deprivation, physical inactivity, or chronic stress may impair osteoblast-mediated bone formation, leading to irreversible deficits in bone mineral density that manifest decades later [6]. Therefore, exploring the important modifiable risk factors that exist during childhood will help promote bone health in adulthood.

Adverse childhood experiences (ACEs) refer to a range of traumatic events that occur during childhood, including child abuse, neglect, and family dysfunction. Numerous studies have shown that individuals with a history of ACEs face a heightened risk of developing adverse physical (e.g., diabetes, cardiovascular disease [CVD]) and psychiatric disorders in middle and old age [7]. Our recent study has suggested that individuals with high ACE exposure not only have an increased prevalence of cardiometabolic diseases in young adulthood but may also have more than twofold increased odds of fracture [8]. Similarly, one animal study highlights the critical role of early life stress in bone homeostasis [9]. Growing evidence indicates that recurrent and extended exposure to severe stress or traumatic experiences can result in the emergence of toxic stress. This condition may lead to the continuous activation of the hypothalamic–pituitary–adrenal (HPA) axis, ultimately causing adverse health outcomes [8,10]. However, despite evidence from human and animal studies supporting a wide range of endocrine dysfunctions that possibly involve endocrine-related axes after ACEs, to date, there is no comprehensive evaluation of the association between ACEs and osteoporosis. Macroscopically, individuals exposed to ACEs are more likely to adopt bone-detrimental behaviors in adulthood (e.g., smoking [OR = 10.4], alcohol abuse [OR = 3.1]) as maladaptive coping mechanisms [11]. Concurrently, chronic stress from ACEs induces HPA axis dysregulation, elevating cortisol levels that directly inhibit osteoblast differentiation and promote osteoclast activity [12,13]. At the molecular level, epigenetic modifications (e.g., DNA hypermethylation) and pro-inflammatory cytokines (e.g., interleukin 6 [IL-6], tumor necrosis factor-alpha [TNF-α]) induced by early-life stress alter bone remodeling equilibrium [14,15,16,17,18]. Therefore, it is reasonable to hypothesize that ACE exposures might increase the risk of osteoporosis.

Crucially, genetic susceptibility may amplify the effect of external risk factors on the risk of osteoporosis [19,20]. This implies that the influence of a specific genetic variation on an individual’s phenotype is contingent upon exposure to external risk factors, a concept referred to as gene–environment (G × E) interaction [21]. However, in prior studies on ACEs, research on the issues of G × E has mainly focused on psychiatric disorders [22,23,24,25], and little is currently known about the combination of genetic predisposition and ACE burden to the risk of osteoporosis. To fill these knowledge gaps, the present study used a large sample of 124,626 adults from the UK Biobank and aimed to examine (1) whether childhood adversity was associated with osteoporosis; (2) test G × E interactions by stratifying genome-wide polygenic risk scores (PRSs) across ACE exposure strata.

## 2. Methods

### 2.1. Study Population

This research utilized data from the UK Biobank cohort, a longitudinal investigation encompassing approximately 0.5 million community-dwelling adults (aged 37–73 years at recruitment) from England, Scotland, and Wales between 2006 and 2010. The cohort employed a population-based recruitment strategy through National Health Service registries, with participants providing digital informed consent prior to completing baseline assessments. These participants completed a wide range of information collection (e.g., sociodemographic, lifestyle, and medical history) through touch-screen or nurse-led questionnaires, anthropometric measurements, and biological sampling. The study protocol and summary characteristics of the included population are available on the UK Biobank website (www.ukbiobank.ac.uk). All participants in the UK Biobank submitted written informed consent. The data collection was conducted in accordance with ethical approval granted by the North West Multi-Centre Research Ethics Committee (approval No. 11/NW/0382, dated: 14 June 2011).

The present study excluded participants with baseline self-reported diagnosis of osteoporosis (N = 10,997). The detailed field ID of these baseline diseases in the UK Biobank is presented in Table S1. This study further excluded participants with missing data on ACEs (N = 340,760), PRS (N = 3701), and covariates (N = 22,154), leaving 124,789 participants remaining in the dataset. Consequently, the complete-case analysis was used in the present study. Post hoc power computations (powerSurvEpi: *powerEpi.default()*) verified sufficient statistical power (>0.90) to detect clinically meaningful associations.

### 2.2. ACE Assessment

The assessment of ACEs was conducted through the Childhood Trauma Screener [26], a shortened version of the Childhood Trauma Questionnaire. This instrument has been shown to be a cost-efficient, valid, and relatively reliable screening tool in large epidemiological studies [26]. It captures five dimensions of early-life adversity: physical abuse, emotional abuse, sexual abuse, physical neglect, and emotional neglect. Participants retrospectively reported exposures prior to age 16 using standardized items including the following: (1) someone physically abused me (physical abuse); (2) I felt that someone in my family hated me (emotional abuse); (3) someone sexually molested me (sexual abuse); (4) there was someone to take me to the doctor if I needed it (physical neglect); and (5) I felt loved (emotional neglect). Responses were recorded on a 5-point Likert scale ranging from “never true” to “very often true”.

Previous studies have validated the thresholds used for the dichotomous classification of each type of ACE [27,28]. Specifically, response thresholds for adversity classification were stratified by category: Neglect domains: (1) physical neglect: “Never”—“Often” responses; (2) emotional neglect: “Never”—“Sometimes” responses; and abuse domains (sexual/physical/emotional): “Rarely”—“Very often” responses. Each affirmed item contributed 1 point to a cumulative adversity index (0–5), where ascending values indicated elevated exposure severity. In the present study, we further stratified participants into three risk tiers: low (no ACEs), intermediate (1–2 ACEs), and high (≥3 ACEs), consistent with thresholds demonstrating dose–response relationships in the prior literature [27,28].

### 2.3. PRS Calculation

Genotyping, imputation, and quality control of the genetic data were carried out by the UK Biobank team. Generally, polygenic risk score (PRS) for osteoporosis could be created using multiple independent single-nucleotide polymorphisms (SNPs), which capture an individual’s load of common genetic variants associated with osteoporosis. These SNPs were reported in previous genome-wide association studies (GWASs) related to osteoporosis. In the present study, the standard PRS for osteoporosis was obtained from Data Field 26,258 in the UK Biobank. In brief, the PRS was generated using a Bayesian approach applied to meta-analyzed (and, when possible, ancestry-specific) summary statistics GWAS data by meta-analyzing multiple external GWAS sources, obtained entirely from external GWAS data. Then, by removing SNPs with low call-rate (<0.05) or MAF < 0.02 in 1000 Genomes, the data were LD-pruned to an approximately independent set of around 185,000 SNPs, and then GWASs were meta-analyzed via fixed-effect inverse variance meta-analysis [29]. A principal component-based ancestry centering step was applied to approximately center the score distributions on zero across all ancestries. Per-individual PRS values were calculated as the genome-wide sum of the per-variant posterior effect size multiplied by allele dosage [29]. For the current study, participants were categorized into three groups (low, intermediate, and high genetic risk) according to the tertile categories of the PRS.

### 2.4. Ascertainment of Outcomes in Adulthood

The outcome of interest investigated was the incidence of osteoporosis, determined according to the 10th version of the International Classification of Diseases (ICD-10). Detailed information regarding the specific ICD-10 codes employed for osteoporosis diagnosis can be found in Supplementary Table S1. For each participant, follow-up was ended upon the earliest event of death, the last recorded date of primary care data, or the diagnosis of new-onset osteoporosis, whichever occurred first.

### 2.5. Covariates

A number of covariates were identified and selected based on the existing literature [30]. In this study, potential covariates included sociodemographic variables (age, sex, ethnicity, and education), socioeconomic variables (Townsend deprivation index [TDI]), lifestyle variables (body mass index [BMI], smoking, alcohol drinking, physical activity, and vitamin/mineral supplement), and comorbidities (diabetes, hypertension, and cardiovascular disease [CVD]). BMI was calculated as the ratio of weight to the square of height. Educational qualifications were coded as a five-factor variable (none; college or university degree; O levels, GCSEs, or CSEs; A levels or AS levels; NVQ, HND, HNC, or other professional), sex as a two-factor variable (women and men), and ethnicity as a two-factor variable (White, non-White). Smoking and alcohol drinking status were coded as a three-factor variable (never, current, and ever). Additionally, physical activity was assessed according to the International Physical Activity Questionnaire and categorized into three groups (low, intermediate, and high). Comorbidities such as hypertension, diabetes mellitus, and CVD were identified through a combination of self-reported diagnostic histories, electronic medical records, medication usage, and clinical assessments. For instance, hypertension was classified based on one or more of the following criteria: self-reported history of hypertension, current use of antihypertensive medications, documentation within electronic medical records indicating hypertension, or measured systolic blood pressure of ≥140 mmHg or diastolic blood pressure of ≥90 mmHg.

### 2.6. Statistical Analyses

Categorical variables were reported as counts and percentages, while continuous variables were presented as means with standard deviations (SDs) for normally distributed data, or as medians with interquartile ranges (IQRs) for data exhibiting skewness. Cox proportional hazard models were used to calculate hazard ratios (HRs) and 95% confidence intervals (CIs) for the associations of incident osteoporosis with ACEs and genetic risk. The proportional hazard assumption was tested using Schoenfeld residuals.

Two models were applied in exploring the effect of ACEs and genetic susceptibility on the risk of osteoporosis. Model 1 provided initial estimates while controlling for age, sex, and ethnicity. In Model 2, estimates were refined by incorporating additional adjustments for BMI, TDI, education, smoking, alcohol drinking, physical activity, vitamin/mineral supplement, diabetes, hypertension, and CVD. Additionally, to explore the sex-specific associations of ACEs and genetic susceptibility with incident osteoporosis, this study further added estradiol in models among female participants, and testosterone was added in models among male participants. Similar modeling strategies were used to explore the association between genetic susceptibility (PRS) and the risk of incident osteoporosis.

Moreover, interactions between ACEs and PRS were tested, incidence rates per 100,000 person-years were calculated, and the joint effects of the combination of genetics and ACEs (9 categories with low genetic risk and low ACE exposure as reference) on incident osteoporosis risk were estimated. Additionally, the current study also performed a sensitivity analysis to explore the joint effects and G × E interactions by restricting analysis to participants of European descent. *p* values below 0.05 were deemed statistically significant. All statistical analyses were conducted using R version 4.4.0 (https://www.r-project.org/).

## 3. Results

The analytical cohort comprised 124,789 participants with a mean age of 55.7 ± 7.8 years (53.8% female). A total of 67,116 (53.8%) participants reported no history of ACEs (low ACEs), 45,975 (36.8%) reported experiencing one or two types of ACEs (intermediate ACEs), and 11,698 (9.4%) reported experiencing three or more types of ACEs (high ACEs). Over a median follow-up of 12.8 years, 2474 incident osteoporosis cases were documented. Comparative analyses demonstrated that osteoporosis cases exhibited significantly higher baseline probabilities of advanced age, female sex, greater socioeconomic deprivation, lower educational attainment, and lower BMI (Table 1). Furthermore, this group showed elevated prevalence rates of physical inactivity and pre-existing diabetes/cardiovascular conditions (all *p* < 0.05).

**Figure 1:**
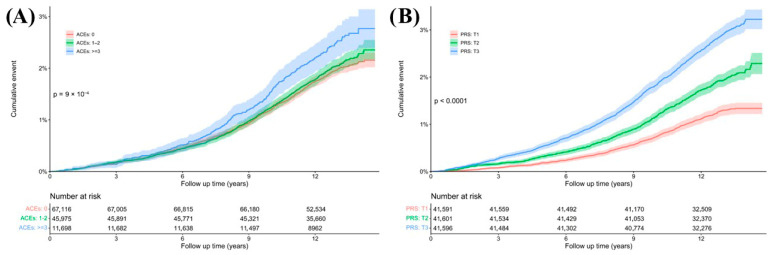
Kaplan–Meier curves for osteoporosis incidence across different ACEs (**A**) and PRS (**B**) categories in the follow-up cohort.

The risk of developing incident osteoporosis exhibited a significant increase across the three categories of ACEs (Table 2 and Table 3 and Figure 1). After adjustments for potential covariates, each one-point rise in ACEs corresponded to a 7% increased risk of developing osteoporosis (HR, 95% CI: 1.07, 1.04–1.11). Individuals with high levels of ACEs exhibited an increased risk of developing incident osteoporosis compared to those with low ACEs (HR, 95% CI: 1.26, 1.10–1.43) (Table 2, Model 2). Moreover, all ACE types examined (abuse/neglect types) were significantly linked to an increased risk of incident osteoporosis (Table 2, Model 2). In the sex-stratified subgroup analysis, the association between high ACEs and the risk of osteoporosis was more pronounced in women (HR, 95% CI:1.28, 1.11–1.48) (Table 3, Model 2), and this association was still significant even after additionally adjusting for estradiol (HR, 95% CI: 1.58, 1.04–2.42) (Table 3, Model 3). Similar results were observed for physical abuse (HR, 95% CI: 1.44, 1.03–2.03).

The risk of osteoporosis significantly increased as the PRS category became more unfavorable (Tables S2 and S3). Participants with a higher PRS (T3) had a significantly increased risk of incident osteoporosis compared to those with a lower PRS (T1) (HR, 95% CI: 2.37, 2.13–2.63). Similar results were observed in women (HR, 95% CI: 2.33, 2.08–2.61) and men (HR, 95% CI: 2.62, 2.00–3.46) participants.

**Figure 2:**
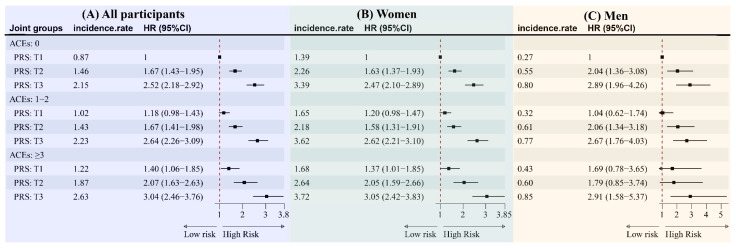
The joint effect of ACEs and the PRS on the incidence of osteoporosis. Note: incidence rate, per 1000 person-year. Abbreviations: ACEs, adverse childhood experiences; PRS, polygenic risk score; HR, hazard ratio; and CI, confidence interval. (**A**) Adjustment for age, ethnicity, BMI, TDI, education, smoking, alcohol drinking, physical activity, vitamin/mineral supplement, diabetes, hypertension, and CVD. (**B**) Adjustment for age, ethnicity, BMI, TDI, education, smoking, alcohol drinking, physical activity, vitamin/mineral supplement, diabetes, hypertension, CVD, and estradiol. (**C**) Adjustment for age, ethnicity, BMI, TDI, education, smoking, alcohol drinking, physical activity, vitamin/mineral supplement, diabetes, hypertension, CVD, and testosterone.

Figure 2 shows the joint effects of ACEs and PRSs on the risk of developing osteoporosis. An increasing trend in osteoporosis risk was noted with higher levels of both ACEs and PRSs. The greatest risk of incident osteoporosis occurred in participants categorized with both a high ACE and PRS. In contrast to participants exhibiting both low ACEs and a low PRS, those with high ACEs and a high PRS demonstrated multivariate-adjusted HRs of 3.04 (95% CIs: 2.46–3.76) for the development of osteoporosis. However, the analysis did not reveal any significant interaction effects between the categories of ACEs and PRS concerning osteoporosis risk (*p* for interaction > 0.05).

After restricting analysis to participants of European descent, the G × E interactions were still insignificant (*p* for interaction > 0.05). Across each category of ACEs—low, intermediate, and high—a notable elevation in the risk of osteoporosis was found among participants with elevated genetic risk compared to their counterparts with lower genetic risk. This heightened risk was particularly evident in individuals categorized with both a high PRS and high ACEs. For instance, the incidence rate was 2.63/1000 person-years, 2.23/1000 person-years, and 2.15/1000 person-years for participants with a high PRS in high, intermediate, and low ACEs, respectively. Similar results were observed in both women and men (Figure 2). Furthermore, similar findings were also observed for each dimension of ACEs or in participants of European descent (Tables S4 and S5).

## 4. Discussion

### 4.1. Key Findings

This population-based large-scale cohort study provided evidence that ACEs were associated with an increased risk of osteoporosis among middle-aged and older adults, and this relationship seems to be more pronounced in women. In addition, when examining the joint effects of genetic risk and ACEs, participants with high ACEs and high genetic risk showed the highest risk of incident osteoporosis, but the G × E interaction did not reach statistical significance.

### 4.2. Comparison with Previous Studies

Mounting evidence suggests that ACEs contribute to lifelong physiological dysregulation, predisposing individuals to cardiometabolic disorders, mental illness, and premature mortality [8,11]. Emerging data extend this paradigm to musculoskeletal health, with childhood adversity linked to suboptimal muscle strength and increased prevalence of sarcopenia or arthritis [31,32,33,34,35]. However, research directly connecting ACEs to osteoporosis remains limited. To date, only two cross-sectional studies have examined the relationship between high ACE levels and increased odds of skeletal fracture in adults [7,8], although these studies are constrained by recall bias and the temporal ambiguity inherent in retrospective designs. Given the established connection between fracture and osteoporosis, these findings might indirectly suggest a link between ACEs and osteoporosis. Additionally, previous studies have demonstrated significantly lower bone mineral density in patients with depressive symptoms compared to those without. Although one study related to ACEs considered osteoporosis as an outcome of interest, it did not report relevant results and instead focused on the analysis of comorbidity count [36]. Similarly, experimental research evidence shows that early-life stress leads to altered bone innervation and a reduction in the expression of some neurogenic and osteogenic mediators involved in bone metabolism [37,38]. These findings also indirectly suggest that early life stress resulting from exposure to various types of ACEs may impair bone health.

To the best of our knowledge, the present prospective cohort study is the first to establish a dose–response relationship between exposure to ACEs (both cumulative burden and specific subtypes) and the incidence of osteoporosis, and this association was pronounced among women. Notably, emotional neglect—an understudied ACE subtype—exhibited comparable risk magnitude to overt abuse (HR: 1.14), highlighting the insidious impact of chronic developmental stressors even in the absence of overt trauma. The present findings extend the limited available evidence on the association between ACEs and the long-term risk of osteoporosis while also providing robust data to support the developmental origin hypothesis of osteoporosis

### 4.3. Potential Mechanisms Underlying the Effects of ACEs

The underlying mechanisms behind the association between ACEs and osteoporosis risk may be linked to toxic stress responses and epigenetic modifications [12,13,39,40,41].

Among the different mechanisms suggested, the toxic stress theory is widely recognized as a key factor in understanding the influence of ACEs on long-term health conditions. Prolonged exposure to intense or repeated adversity during childhood can trigger a toxic stress response, which results in persistent activation of the HPA axis. This overactivation leads to the continuous release of high cortisol levels into the bloodstream. Elevated cortisol, in turn, inhibits osteoblast differentiation and promotes osteoclast activity [12,13], ultimately increasing the risk of osteoporosis. It is important to note that, in women, elevated cortisol levels may also suppress estrogen production by interfering with ovarian function and altering estrogen metabolism in the liver [39], and this could potentially explain why the association between ACEs and osteoporosis is more pronounced in the female population in this study.

In addition to toxic stress, epigenetics provides another important avenue for understanding the long-term effects of ACEs. Epigenetics primarily serve as post-transcriptional regulators and play crucial roles within the biological signaling regulatory network, and studies have shown that epigenetic mechanisms are intimately linked to processes such as osteogenic differentiation, osteogenesis, bone remodeling, and other aspects of bone metabolism [42]. Epigenetic studies indicate that environmental factors, including ACEs, can modify gene expression and accelerate cellular aging through multiple pathways [40,41]. The childhood period is particularly critical, as this period is when epigenetic modifications are most impactful [43]. Adverse experiences during this stage, such as psychological or physical abuse and neglect, may influence the expression of genes related to bone development and maintenance through DNA methylation [44]. Importantly, these epigenetic modifications create a biological “memory” that may have lasting health consequences throughout an individual’s life [40]. However, the present study did not assess biological indicators that underpin the relationship between ACEs and osteoporosis; therefore, it is essential for future research to investigate the specific mechanisms.

Notably, while the present study hypothesized that genetic susceptibility might exacerbate the osteoporotic risk associated with ACEs, current analyses revealed no statistically significant interaction effects between ACE exposure and PRS (*p*-interaction > 0.05). This observation suggests that ACEs and genetic predisposition may independently contribute to osteoporosis pathogenesis through distinct biological pathways, rather than exhibiting synergistic effects. Nevertheless, the substantially elevated risk observed in the high ACEs/high-PRS group (HR: 3.04; 95% CI: 2.46–3.76) compared to the low ACEs/low-PRS reference group reinforces the clinical importance of considering both early-life adversity and genetic loading in risk stratification. A critical caveat arises from the limited ancestral diversity in the current cohort, where 94.6% of participants were of European descent. Future multi-ethnic studies are warranted to determine whether this additive risk paradigm extends to populations with differing genetic architectures and socioenvironmental contexts.

### 4.4. Public Health Implications

The present findings have significant public health implications, especially in improving the prevention and management of osteoporosis. First, while we acknowledge the logistical, ethical, and policy challenges in integrating ACEs into clinical and public health strategies, we believe that incorporating ACEs into fracture risk evaluation tools could still enhance osteoporosis prevention efforts, particularly for women. Recognizing the long-term impact of ACEs on bone health could lead to more personalized and effective risk stratification, helping clinicians identify individuals at higher risk for osteoporosis earlier. Second, while the contribution of ACEs to osteoporosis incidence may appear relatively modest (estimated at 2.4% of cases), the potential for early intervention remains a crucial aspect of this approach. Youth exposed to ACEs should be targeted for comprehensive preventive strategies, including nutritional support (such as calcium and vitamin D supplementation), weight-bearing exercise programs, and stress-reduction therapies [45,46]. These interventions can help mitigate latent bone loss and improve bone health outcomes before osteoporosis develops [47,48]. By addressing the roots of the problem early on, it might reduce the long-term health burden associated with ACEs. Finally, policy advocacy plays a vital role in reducing the incidence of ACEs and their subsequent impact on bone health. Strengthening childhood social safety nets, such as anti-bullying policies and parental health education, can create a supportive environment that minimizes childhood adversities. The combination of ACE-based risk stratification with other modifiable risk factors could provide a more comprehensive, personalized approach to osteoporosis prevention.

### 4.5. Limitations of This Study

This study possesses several notable strengths. First, the extensive sample size enabled the investigation of potential modification effects attributable to genetic predisposition while simultaneously ensuring adequate statistical power for these analyses. Second, this study used a single well-respected nationwide project with reliable data collection. Despite its strengths, we also recognize that it is not without limitations. First, as there may be specific sensitive exposure windows from birth to adulthood [44], this study only evaluated ACEs occurring before the age of 16, which represents a relatively broad time span. Second, similar to many prior studies on ACEs, there is a potential for recall bias, as data were gathered through self-reported questionnaires, particularly among individuals of higher age. Third, a large number of participants were excluded from the present study due to missing data on exposure variables, outcome variables, or covariates. This potential selection bias may have impacted the generalizability of the results. Finally, although multivariate adjustments incorporated comprehensive confounder profiles, we were unable to eliminate the possibility of residual confounding arising from unmeasured or unavailable covariates.

## 5. Conclusions

In conclusion, this study provides robust evidence that childhood adversities contribute to an increased risk of osteoporosis, independent of genetic predisposition. Specifically, this study indicates that cumulative ACE burden, particularly emotional neglect, emotional abuse, physical abuse, and sexual abuse, is associated with an elevated risk of osteoporosis, with a dose–response relationship observed. These findings suggest the importance of incorporating childhood adversity screening into osteoporosis prevention strategies and highlight the need for targeted bone health interventions for youth exposed to childhood adversities.

## Figures and Tables

**Table 1:** Baseline characteristics of the study population (N = 124,789).

Characteristics	Participants Without Osteoporosis	Participants with Osteoporosis	*p*
ACEs, n (%)			0.0013
0	65,839 (53.8)	1277 (51.6)	
1–2	45,060 (36.8)	915 (37)	
≥3	11,416 (9.3)	282 (11.4)	
Age, years	55.6 (7.8)	59.9 (6.2)	<0.001
Sex, n (%)			<0.001
female	65,099 (53.2)	2063 (83.4)	
male	57,216 (46.8)	411 (16.6)	
BMI, kg/m^2^	26.8 (4.5)	25.5 (4.6)	<0.001
Ethnicity, n (%)			0.808
non-white	9847 (8.1)	203 (8.2)	
white	112,468 (91.9)	2271 (91.8)	
TDI	−1.7 (2.8)	−1.5 (2.9)	0.001
Educational qualifications, n (%)			<0.001
College or university degree	58,418 (47.8)	1020 (41.2)	
A levels/AS levels or equivalent	16,750 (13.7)	335 (13.5)	
O levels/GCSEs or equivalent	23,536 (19.2)	534 (21.6)	
CSEs or equivalent	4287 (3.5)	76 (3.1)	
NVQ or HND or HNC or equivalent	6172 (5)	111 (4.5)	
Other professional qualifications e.g., nursing, teaching	5860 (4.8)	167 (6.8)	
None of the above	7292 (6)	231 (9.3)	
Smoking, n (%)			0.029
never	70,301 (57.5)	1366 (55.2)	
previous	43,163 (35.3)	937 (37.9)	
current	8851 (7.2)	171 (6.9)	
Drinking, n (%)			<0.001
never	3189 (2.6)	97 (3.9)	
previous	3106 (2.5)	109 (4.4)	
current	116,020 (94.9)	2268 (91.7)	
Physical activity, n (%)			0.028
low	22,389 (18.3)	498 (20.1)	
moderate	52,687 (43.1)	1072 (43.3)	
high	47,239 (38.6)	904 (36.5)	
Supplement, n (%)			<0.001
no	118,838 (97.2)	2326 (94)	
yes	3477 (2.8)	148 (6)	
Diabetes, n (%)			0.014
no	91,033 (74.4)	1797 (72.6)	
Borderline	25,452 (20.8)	530 (21.4)	
yes	5830 (4.8)	147 (5.9)	
Hypertension, n (%)			0.677
no	58,881 (48.1)	1180 (47.7)	
yes	63,434 (51.9)	1294 (52.3)	
Self-reported CVD, n (%)			<0.001
no	117,034 (95.7)	2330 (94.2)	
yes	5281 (4.3)	144 (5.8)	

**Table 2:** The longitudinal associations of exposure to ACEs with osteoporosis.

ACEs	Model 1 ^a^ (HR [95%CI])	Model 2 ^b^ (HR [95%CI])
Emotional abuse	1.17 (1.05–1.30)	1.14 (1.03–1.27)
Physical abuse	1.15 (1.03–1.27)	1.17 (1.05–1.30)
Sexual abuse	1.15 (1.01–1.31)	1.15 (1.01–1.31)
Emotional neglect	1.17 (1.07–1.28)	1.14 (1.04–1.25)
Physical neglect	1.12 (1.01–1.23)	1.11 (1.00–1.23)
ACE score, each unit increases	1.08 (1.04–1.11)	1.07 (1.04–1.11)
ACE score: 0	ref	ref
ACE score: 1–2	1.07 (0.98–1.16)	1.06 (0.98–1.16)
ACE score: ≥3	1.28 (1.13–1.46)	1.26 (1.10–1.43)

Note: Abbreviations: ACEs, adverse childhood experiences; HR, hazard ratio; and CI, confidence interval. ^a^ adjustment for age, sex, and ethnicity, ^b^ adjustment for age, sex, ethnicity, BMI, TDI, education, smoking, alcohol drinking, physical activity, vitamin/mineral supplement, diabetes, hypertension, and CVD.

**Table 3:** Sex-specific associations of ACEs with osteoporosis.

ACEs	Women (HR [95%CI])			Men (HR [95%CI])		
	Model 1 ^a^	Model 2 ^b^	Model 3 ^c^	Model 1 ^a^	Model 2 ^b^	Model 3 ^d^
emotional abuse	1.16 (1.03–1.30)	1.14 (1.02–1.28)	1.19 (0.83–1.69)	1.23 (0.93–1.64)	1.13 (0.84–1.51)	1.03 (0.75–1.40)
physical abuse	1.16 (1.03–1.30)	1.19 (1.06–1.34)	1.44 (1.03–2.03)	1.11 (0.87–1.41)	1.08 (0.85–1.37)	1.12 (0.87–1.43)
sexual abuse	1.18 (1.04–1.35)	1.20 (1.05–1.37)	1.35 (0.89–2.04)	0.90 (0.58–1.38)	0.82 (0.53–1.26)	0.82 (0.52–1.29)
emotional neglect	1.19 (1.08–1.32)	1.17 (1.06–1.29)	1.17 (0.84–1.62)	1.06 (0.84–1.34)	0.98 (0.77–1.24)	0.96 (0.75–1.23)
physical neglect	1.08 (0.97–1.20)	1.08 (0.97–1.20)	0.96 (0.64–1.43)	1.33 (1.05–1.69)	1.24 (0.97–1.59)	1.17 (0.90–1.51)
ACEs score, each unit increases	1.08 (1.04–1.12)	1.08 (1.04–1.12)	1.10 (0.98–1.24)	1.08 (0.99–1.19)	1.04 (0.95–1.14)	1.02 (0.93–1.13)
ACEs score: 0	ref	ref	ref	ref	ref	ref
ACEs score: 1–2	1.07 (0.97–1.17)	1.07 (0.97–1.17)	1.12 (0.82–1.55)	1.06 (0.87–1.30)	1.03 (0.84–1.26)	0.98 (0.79–1.21)
ACEs score: ≥3	1.28 (1.12–1.47)	1.28 (1.11–1.48)	1.58 (1.04–2.42)	1.28 (0.89–1.83)	1.11 (0.77–1.60)	1.08 (0.74–1.58)

Note: Abbreviations: ACEs, adverse childhood experiences; HR, hazard ratio; and CI, confidence interval. ^a^ adjustment for age and ethnicity, ^b^ adjustment for age, ethnicity, BMI, TDI, education, smoking, alcohol drinking, physical activity, vitamin/mineral supplement, diabetes, hypertension, and CVD. ^c^ adjustment for age, ethnicity, BMI, TDI, education, smoking, alcohol drinking, physical activity, vitamin/mineral supplement, diabetes, hypertension, CVD, and estradiol. ^d^ adjustment for age, ethnicity, BMI, TDI, education, smoking, alcohol drinking, physical activity, vitamin/mineral supplement, diabetes, hypertension, CVD, and testosterone.

## Data Availability

Study protocol: Not applicable. Statistical code: May be made available upon reasonable request (e-mail, mingyangwu@csu.edu.cn). Dataset: Data from the UK Biobank are accessible to researchers via application to the UK Biobank (www.ukbiobank.ac.uk).

## Supplementary Materials

The following supporting information can be downloaded at https://www.mdpi.com/article/10.3390/medicina61081387/s1. Table S1-1: UK Biobank Data-Field IDs used in the study and types of variables included in the models; Table S1-2: Disease definition; Table S2: The relationship between PRS and the risk of osteoporosis; Table S3: Sex-specific relationships between PRS and the risk of osteoporosis; Table S4: The joint effect of ACEs and PRS on the risk of osteoporosis; Table S5: The joint effect of cumulative ACEs exposure and PRS on the risk of osteoporosis in participants of European descent.

## Abbreviations

The following abbreviations are used in this manuscript:

ACEs	adverse childhood experiences
PRS	polygenic risk score
CVD	cardiovascular disease
SNPs	single-nucleotide polymorphisms
GWASs	genome-wide association studies
BMI	body mass index
TDI	Townsend deprivation index
HRs	hazard ratios
HPA	hypothalamic–pituitary–adrenal
CI	confidence interval
IQR	interquartile range
SD	standard deviation
ICD	International Classification of Diseases
IL-6	interleukin 6
TNF-α	tumor necrosis factor-alpha
