# Supplementary material for: Adverse Childhood Experiences, Genetic Susceptibility, and the Risk of Osteoporosis: A Cohort Study
Source: Medicina (Kaunas). 2025 Jul 30;61(8):1387. doi: 10.3390/medicina61081387 (PMC12388128; doi:10.3390/medicina61081387)
Supplement: Supplementary file 1 [file medicina-61-01387-s001.zip › medicina-3679335-supplementary.pdf]

Adverse childhood experiences, genetic susceptibility, and the risk of osteoporosis: a cohort study

Yanling Shu [1,2,†], Chao Tu [3,4,†], Yunyun Liu [5], Lulu Song [1,2], Youjie Wang [1,2], Mingyang Wu [6,*]

[1] Ministry of Education Key Laboratory of Environment and Health, and State Key Laboratory of Environmental Health (Incubating), School of Public Health, Tongji Medical College, Huazhong University of Science and Technology, No.13 Hangkong Road, Wuhan, China, 430030;

[2] Department of Maternal and Child Health, School of Public Health, Tongji Medical College, Huazhong University of Science and Technology, No.13 Hangkong Road, Wuhan, China, 430030;

[3] Department of Orthopaedics, The Second Xiangya Hospital of Central South University, Changsha, Hunan 410011, China;

[4] Hunan Key Laboratory of Tumor Models and Individualized Medicine, Hunan Engineering Research Center of AI Medical Equipment, The Second Xiangya Hospital of Central South University, Changsha, Hunan 410011, China

[5] School of Medical Technology, Jiangsu College of Nursing, Huai'an, Jiangsu, China

[6] Department of Maternal and Child Health, Xiangya School of Public Health, Central South University, No.172 Tongzipo Road, Changsha 410013, China

[†] Yanling Shu and Chao Tu made equal contributions to this manuscript

[*] **Corresponding Author:**

Mingyang Wu,

E-mail: mingyangwu@csu.edu.cn,

ORCID: 0000-0003-3001-1897

Address: Xiangya School of Public Health, Central South University, 172 Tongzipo Road, Yuelu District, Changsha City, Hunan Province, China 410013.

Table S1-1 UK Biobank Data-Field IDs used in the study and types of variables included in the models.

**Covariates**	**Data-Field**	**Types of Variables**
Age	21022	Continuous
Ethnicity	21000	Categorical
Deprivation index	189	Continuous
Current smokers	1239	Categorical
Alcohol intake	1558	Continuous
Body-mass index	21001	Continuous
Physical activity	22032	Categorical
Vitamin D supplements	20003	Categorical
Calcium supplements	20003	Categorical
Cardiovascular disease	20001, 20002	Categorical
Hypertension	20001, 20002	Categorical
Diabetes	20001, 20002	Categorical
emotional abuse	20487	Categorical
physical abuse	20488	Categorical

sexual abuse	20490	Categorical
emotional neglect	20489	Categorical
physical neglect	20491	Categorical
PRS for osteoporosis	26258	Continuous

Table S1-2 Disease definition.

**ICD-10 Code for Osteoporosis**	**Description**
Osteoporosis with pathological fracture	
M80	Osteoporosis with pathological fracture
M80.0	Postmenopausal osteoporosis with pathological fracture
M80.1	Postoophorectomy osteoporosis with pathological fracture
M80.2	Osteoporosis of disuse with pathological fracture
M80.3	Postsurgical malabsorption osteoporosis with pathological fracture
M80.4	Drug-induced osteoporosis with pathological fracture
M80.5	Idiopathic osteoporosis with pathological fracture
M80.8	Other osteoporosis with pathological fracture

M80.9	Unspecified osteoporosis with pathological fracture
Osteoporosis without pathological fracture	
M81.0	Postmenopausal osteoporosis
M81.1	Postoophorectomy osteoporosis
M81.2	Osteoporosis of disuse
M81.3	Postsurgical malabsorption osteoporosis
M81.4	Drug-induced osteoporosis
M81.5	Idiopathic osteoporosis
M81.6	Localized osteoporosis [Lequesne]
M81.8	Other osteoporosis
M81.9	Osteoporosis, unspecified
Osteoporosis in diseases classified elsewhere	
M82.0	Osteoporosis in multiple myelomatosis
M82.1	Osteoporosis in endocrine disorders
M82.8	Osteoporosis in other diseases classified elsewhere

Table S2 The relationship between PRS and the risk of osteoporosis

	Model1[a] (HR [95%CI])	Model2[b] (HR [95%CI])
PRS, each unit increases	1.52 (1.45-1.58)	1.51 (1.44-1.57)
T1	ref	ref
T2	1.56 (1.40-1.75)	1.55 (1.38-1.73)
T3	2.39 (2.16-2.66)	2.37 (2.13-2.63)

Note: Abbreviations: PRS, polygenic risk scores, HR, hazard ratio, CI, confidence interval.

[a] adjustment for age, sex, and ethnicity,

[b] adjustment for age, sex, ethnicity, BMI, TDI, education, smoke, alcohol drink, physical activity, vitamin/mineral supplement, diabetes, hypertension, and CVD.

Table S3 Sex-specific relationships between PRS and the risk of osteoporosis

PRS	Women (HR [95%CI])			Men (HR [95%CI])		
	Model1[a]	Model2[b]	Model3[c]	Model1[a]	Model2[b]	Model3[d]
PRS, each unit increases	1.50 (1.43-1.57)	1.49 (1.42-1.56)	1.48 (1.27-1.73)	1.61 (1.45-1.79)	1.61 (1.45-1.78)	1.59 (1.43-1.77)
T1	ref	ref	ref	ref	ref	ref
T2	1.51 (1.33-1.70)	1.49 (1.32-1.68)	1.59 (1.05-2.40)	1.89 (1.42-2.51)	1.88 (1.41-2.49)	1.90 (1.42-2.55)
T3	2.35 (2.10-2.63)	2.33 (2.08-2.61)	2.50 (1.70-3.68)	2.64 (2.02-3.46)	2.62 (2.00-3.43)	2.63 (1.99-3.48)

Note: Abbreviations: PRS, polygenic risk scores, HR, hazard ratio, CI, confidence interval.

[a] adjustment for age and ethnicity,

[b] adjustment for age, ethnicity, BMI, TDI, education, smoke, alcohol drink, physical activity, vitamin/mineral supplement, diabetes, hypertension, and CVD.

[c] adjustment for age, ethnicity, BMI, TDI, education, smoke, alcohol drink, physical activity, vitamin/mineral supplement, diabetes, hypertension, CVD, and estradiol.

[d] adjustment for age, ethnicity, BMI, TDI, education, smoke, alcohol drink, physical activity, vitamin/mineral supplement, diabetes, hypertension, CVD, and testosterone.

Table S4 The joint effect of ACEs and PRS on the risk of osteoporosis

ACEs	Categories	Model 1[a] (HR [95%CI])	Model 2[b] (HR [95%CI])
Emotional abuse	Emotional abuse: no		
	PRS: T1	ref	ref
	PRS: T2	1.60 (1.41-1.80)	1.58 (1.40-1.79)
	PRS: T3	2.47 (2.20-2.77)	2.45 (2.18-2.75)
	Emotional abuse: yes		
	PRS: T1	1.32 (1.06-1.66)	1.30 (1.04-1.63)
	PRS: T2	1.87 (1.53-2.27)	1.80 (1.48-2.19)
	PRS: T3	2.72 (2.29-3.23)	2.63 (2.21-3.12)
Physical abuse	Physical abuse: no		
	PRS: T1	ref	ref
	PRS: T2	1.56 (1.38-1.76)	1.54 (1.37-1.74)
	PRS: T3	2.34 (2.08-2.62)	2.32 (2.07-2.60)
	Physical abuse: yes		
	PRS: T1	1.06 (0.84-1.34)	1.08 (0.86-1.38)
	PRS: T2	1.69 (1.39-2.05)	1.70 (1.40-2.07)
	PRS: T3	2.85 (2.42-3.36)	2.86 (2.42-3.37)
Sexual abuse	Sexual abuse: no		
	PRS: T1	ref	ref
	PRS: T2	1.59 (1.41-1.79)	1.58 (1.40-1.77)
	PRS: T3	2.41 (2.16-2.70)	2.39 (2.14-2.67)
	Sexual abuse: yes		
	PRS: T1	1.25 (0.96-1.64)	1.25 (0.96-1.64)
	PRS: T2	1.69 (1.34-2.15)	1.67 (1.32-2.12)
	PRS: T3	2.82 (2.32-3.43)	2.78 (2.28-3.38)
Emotional neglect	Emotional neglect: no		
	PRS: T1	ref	ref
	PRS: T2	1.59 (1.40-1.81)	1.58 (1.39-1.79)
	PRS: T3	2.44 (2.16-2.75)	2.42 (2.14-2.73)
	Emotional neglect: yes		
	PRS: T1	1.24 (1.01-1.51)	1.21 (0.99-1.48)
	PRS: T2	1.83 (1.54-2.18)	1.76 (1.48-2.10)
	PRS: T3	2.80 (2.40-3.26)	2.68 (2.30-3.13)
Physical neglect	Physical neglect: no		
	PRS: T1	ref	ref
	PRS: T2	1.65 (1.45-1.87)	1.63 (1.44-1.85)
	PRS: T3	2.56 (2.28-2.89)	2.54 (2.25-2.86)
	Physical neglect: yes		

	PRS: T1	1.40 (1.14-1.72)	1.39 (1.13-1.71)
	PRS: T2	1.80 (1.50-2.17)	1.77 (1.47-2.14)
	PRS: T3	2.60 (2.20-3.06)	2.55 (2.16-3.01)
ACEs score	ACES: 0		
	PRS: T1	ref	ref
	PRS: T2	1.69 (1.44-1.98)	1.67 (1.43-1.95)
	PRS: T3	2.54 (2.19-2.94)	2.52 (2.18-2.92)
	ACES: 1-2		
	PRS: T1	1.19 (0.99-1.44)	1.18 (0.98-1.43)
	PRS: T2	1.68 (1.42-2.00)	1.67 (1.41-1.98)
	PRS: T3	2.69 (2.31-3.15)	2.64 (2.26-3.09)
	ACES: >=3		
	PRS: T1	1.41 (1.06-1.86)	1.40 (1.06-1.85)
	PRS: T2	2.15 (1.69-2.73)	2.07 (1.63-2.63)
	PRS: T3	3.14 (2.54-3.88)	3.04 (2.46-3.76)

Note: Abbreviations: ACEs, adverse childhood experiences, PRS, polygenic risk scores, HR, hazard ratio, CI, confidence interval.

[a] adjustment for age, sex, and ethnicity,

[b] adjustment for age, sex, ethnicity, BMI, TDI, education, smoke, alcohol drink, physical activity, vitamin/mineral supplement, diabetes, hypertension, and CVD.

Table S5 The joint effect of cumulative ACEs exposure and PRS on the risk of osteoporosis in participants of European descent

	Model 1[a] (HR [95%CI])	Model 2[b] (HR [95%CI])
ACES: 0		
PRS: T1	ref	ref
PRS: T2	1.69 (1.44-1.98)	1.67 (1.43-1.95)
PRS: T3	2.54 (2.19-2.94)	2.52 (2.18-2.92)
ACES: 1-2		
PRS: T1	1.20 (0.99-1.44)	1.18 (0.98-1.43)
PRS: T2	1.69 (1.42-2.00)	1.67 (1.41-1.98)
PRS: T3	2.70 (2.31-3.15)	2.65 (2.27-3.09)
ACES: >=3		
PRS: T1	1.41 (1.07-1.87)	1.40 (1.06-1.86)
PRS: T2	2.16 (1.70-2.75)	2.08 (1.63-2.64)
PRS: T3	3.16 (2.55-3.90)	3.05 (2.46-3.77)

Note: Abbreviations: ACEs, adverse childhood experiences, PRS, polygenic risk scores, HR, hazard ratio, CI, confidence interval.

[a] adjustment for age, sex, and ethnicity,

[b] adjustment for age, sex, ethnicity, BMI, TDI, education, smoke, alcohol drink, physical activity, vitamin/mineral supplement, diabetes, hypertension, and CVD.
